# Effect of Graphene Oxide Quantities on Microhardness of Cured-Surface Coating Agents

**DOI:** 10.3390/polym17111472

**Published:** 2025-05-26

**Authors:** Khanaphan Lebkrut, Awiruth Klaisiri, Somporn Swasdison, Niyom Thamrongananskul, Somphob Thompho, Tool Sriamporn

**Affiliations:** 1 Department of Prosthodontics, Faculty of Dentistry, Chulalongkorn University, Bangkok 10330, Thailand; 6571001932@student.chula.ac.th (K.L.); niyom.t@chula.ac.th (N.T.); 2 Division of Restorative and Esthetic Dentistry, Faculty of Dentistry, Thammasat University, Pathum Thani 12120, Thailand; 3 Department of Oral Medicine, College of Dental Medicine, Rangsit University, Pathum Thani 12000, Thailand; somporn.s@rsu.ac.th; 4 Faculty of Pharmaceutical Sciences, Chulalongkorn University, Bangkok 10330, Thailand; somphob.t@chula.ac.th; 5 Department of Prosthodontics, College of Dental Medicine, Rangsit University, Pathum Thani 12000, Thailand

**Keywords:** graphene, graphene family nanomaterials, graphene oxide, microhardness, surface coating agents

## Abstract

This study aimed to investigate the impact of varying concentrations of graphene oxide (GO) combined with two surface coating agents (SCAs) and two dental adhesives (DAHs) used as SCAs on microhardness. Two SCAs, Resin Glaze (ReG) and Coat-It (CoI) (Shofu Inc., Kyoto, Japan), along with two DAHs, Adper^TM^ Scotchbond^TM^ Multi-purpose Adhesive (AdA) (3M ESPE, Seefeld, Germany) and OptiBond^TM^ FL Adhesive (OpA) (Kuraray Noritake Dental Inc., Okayama, Japan), were tested. The ten concentrations of GO—0 wt % (control), 0.05 wt %, 0.1 wt %, 0.3 wt %, 0.5 wt %, 0.7 wt %, 1 wt %, 2 wt %, 5 wt %, and 10 wt %—were incorporated into the SCAs and DAHs to create the experimental formulations. These mixtures underwent centrifugation for homogenization, followed by sonication for dispersion. The mixture was poured into the 3D-printed resin mold (10 mm in diameter and 1 mm in height) and then cured with a light curing unit for 180 s. The cured specimens were then kept in distilled water at 37 ± 1 °C for 24 h. All specimens were then subjected to evaluation of their microhardness properties using a Knoop hardness testing machine. Data were collected, and the statistical analysis was conducted using Two-way ANOVA followed by Tukey’s post-hoc tests at a 0.05 level of significance. According to the results, surface hardness was significantly increased (*p* < 0.05) when 0.3–0.7 wt % of GO was added to ReG, CoI, and AdA, compared to the control group. However, surface hardness was significantly increased (*p* < 0.05) when 0.05–0.3 wt % of GO was added to OpA compared to the control group. In the control groups, the microhardness of OpA was significantly higher than that of the other groups (*p* < 0.05). In the 0.1 wt % groups, the microhardness of OpA was significantly higher than that of the other groups (*p* < 0.05). At 0.5 wt %, ReG, CoI, and AdA showed significantly higher microhardness compared to their respective control groups (*p* < 0.05). In the 1–10 wt % groups, the microhardness of ReG, CoI, and AdA demonstrated a gradual, significant decrease compared to the 0.7 wt % groups. Whereas in the 0.5–10 wt % groups, the microhardness of OpA showed a significant gradual decrease compared to the 0.3 wt % group. In summary, the optimal GO concentration could improve the surface hardness of ReG, CoI, AdA, and OpA.

## 1. Introduction

Provisional restorations are essential in both restorative and cosmetic dentistry, providing temporary solutions to preserve dental health and function. They are particularly important in fixed prosthodontic treatments, where long-term functional needs or further interventions are expected. For provisional restorations to be effective as temporary replacements, they must possess a range of key characteristics. These include strong mechanical properties such as proper fit, stability, rigidity, and resistance to chewing forces. They also need to fulfill biological requirements such as low water absorption, biocompatibility, and resistance to degradation and wear [1,2,3,4,5]. Provisional restorations should have a smooth surface finish to reduce the likelihood of microbial accumulation on the restoration and adjacent oral structures [6,7,8,9]. Therefore, a reduction in porosity can enhance the mechanical properties of the material [10]. Mechanical and chemical polishing methods are techniques employed to reduce surface imperfections such as scratches and porosity [11]. A reduction in porosity directly correlates with enhanced mechanical properties [10]. Likewise, temporary prostheses are subject to meticulous finishing and polishing techniques, employing coarse and fine abrasives to achieve optimal surface smoothness. Surface coating agents (SCAs) have been developed to mitigate the formation of scratches and porosity on provisional restorations [12,13,14,15,16]. Moreover, these coatings contribute to enhanced mechanical properties, resulting in the increased strength of the provisional materials while also effectively preventing the prostheses from discoloration during function [17]. Currently, dental adhesives (DAHs)—which are traditionally employed to bond dental materials—can be used as a substrate for surface coatings. Despite their potential as a surface coating substrate, DAHs frequently lack adequate mechanical strength to withstand the wear and tear associated with occlusal stress. To improve the mechanical properties of DAHs, a number of studies have investigated the addition of various fillers [18,19,20,21]. Different types of nanofillers can be added to dental restorative resins to improve their performance. Metal oxide nanoparticles, such as silicon dioxide, titanium dioxide, and zirconium dioxide, are particularly effective at enhancing mechanical properties such as wear resistance, flexural strength, and tensile strength, which, in turn, increases the durability of restorations [22,23,24,25,26]. Previous research has explored the influence of various nanofillers on the mechanical properties of resin-based materials [27,28,29,30].

Since the discovery of graphene in 2004, various methods for producing graphene layers and thin films have been developed [31]. Graphene, a single layer of carbon atoms arranged in a hexagonal lattice, is the thinnest and strongest material known [32], with a carbon-carbon bond length of 0.142 nanometers [33]. It exhibits remarkable properties such as high electrical conductivity, transparency, biocompatibility, and mechanical strength—being over 100 times stronger than steel by weight [33]. These exceptional characteristics have led to the widespread use of graphene and its derivatives in various scientific and technological fields [34,35]. In dentistry, the application of graphene has been extended to coatings for dental implants [36], tissue engineering [37], and enhancing material properties. It has been shown to improve the hardness, flexural strength, compressive strength, and tensile strength of dental materials [38,39,40]. Additionally, graphene’s mechanical and tribological properties offer significant benefits, contributing to more durable and effective dental restorations [41].

Graphene-based materials have shown great potential as fillers for dental materials, with numerous studies confirming their ability to enhance the mechanical properties of both temporary and final prostheses. Previous studies have shown that even small differences in graphene concentration can significantly affect the mechanical properties of dental materials [42,43,44,45]. However, there is limited research specifically examining the integration of graphene into DAHs and its effect on surface hardness, particularly regarding the optimal concentration for significant improvement in mechanical properties. This study aims to investigate how varying concentrations of graphene oxide (GO) combined with SCAs influence the microhardness of dental materials. The null hypothesis is that there is no statistically significant difference in the mechanical properties of SCAs containing varying concentrations of GO filler.

## 2. Materials and Methods

### 2.1. Graphene Oxide Synthesis

Graphite powder was oxidized using a modified Hummers’ method [46]. This involved mixing graphite powder (3 g) and potassium permanganate (KMnO_4_) (9 g) and cooling the mixture to 0 °C. Sulfuric acid (H_2_SO_4_) (150 mL) was then added while maintaining a temperature below 15 °C. The temperature was gradually increased to 95 °C over an hour while adding distilled water (150 mL) under constant stirring. Thirty percent hydrogen peroxide (H_2_O_2_) (30 mL) and distilled water were added to the reaction mixture until it turned yellow-brown. The resulting GO nanosheets were separated via centrifugation and washed with hydrochloric acid (HCl) solution to remove sulfate ions. The purified nanosheets were filtered and rinsed with distilled water until a neutral pH was achieved. The mixture was vacuum-dried at 65 °C for 24 h to turn it into GO powder.

### 2.2. Graphene Oxide Modified in Adhesive

Two commercial SCAs and DAHs were selected: Resin Glaze (ReG), Coat-It (CoI), Adper^TM^ Scotchbond^TM^ Multi-purpose Adhesive (AdA), and OptiBond^TM^ FL Adhesive (OpA) (Table 1). The unaltered adhesives were regarded as controls. Nine concentrations of GO—0.05 wt %, 0.1 wt %, 0.3 wt %, 0.5 wt %, 0.7 wt %, 1 wt %, 2 wt %, 5 wt %, and 10 wt %—were added to the respective SCAs to synthesize experimental GO adhesives. A precision electronic weighing balance (AS220/C/2, Radwag, Radom, Poland) was used to accurately identify the percentage of GO weight. GO powder was added to the ethanol solvent, and the mixture was prepared using magnetic stirrers (HI190M-2, Hanna, Cluj Napoca, Romania) and a condenser (SA300; Sansyo, Tokyo, Japan). The experimental adhesives were centrifuged to achieve a homogeneous dispersion of GO, followed by sonication for one hour to ensure even mixing. To prevent photo-polymerization due to ambient light, the mixtures were covered with foil and mixed under a red light in a dark box. Due to the adhesive’s short shelf life, it was kept at 4 °C and utilized within two weeks. Furthermore, to eliminate the ethanol solvent, the mixtures underwent slow nitrogen air drying at a controlled airflux pressure of 0.2 bar (according to the reference commercial adhesive datasheet).

**Figure 1:**
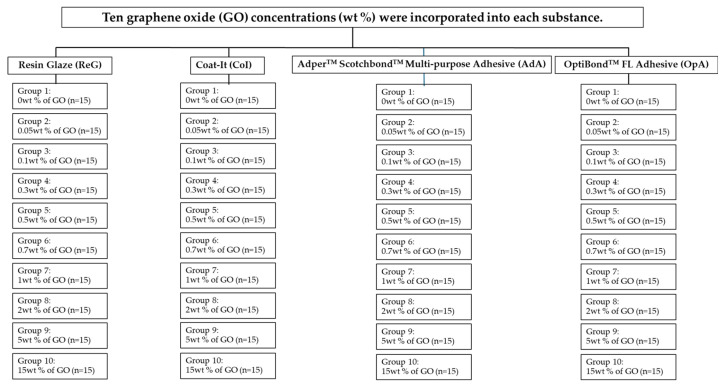
Flow chart of specimens’ preparation for the hypothesis testing.

Specimens were prepared using a 3D-printed resin mold, 10 mm in diameter and 1 mm in height. The control and experimental GO adhesives were then poured into the resin molds and coated with a piece of Mylar. Specimens were prepared in a nitrogen container to prevent the formation of an oxygen-inhibited layer. Following the manufacturer’s instructions, the specimens were polymerized for 180 s. The AdA, OpA, and CoI groups were polymerized using an LED curing unit (Elipar DeepCure-S, 3M ESPE, Seefeld, Germany) with a wavelength of 440–490 nm, while the ReG group was polymerized with an LED curing unit (Bluephase N^®^, Ivoclar Vivadent AG, Schaan, Liechtenstein) with a wavelength of 400–410 nm. All specimens were stored in distilled water at 37 ± 1 °C for 24 h before surface microhardness testing. Figure 1 shows a flow chart of the specimens’ preparation for the hypothesis testing.

### 2.3. Microhardness Test

**Figure 2:**
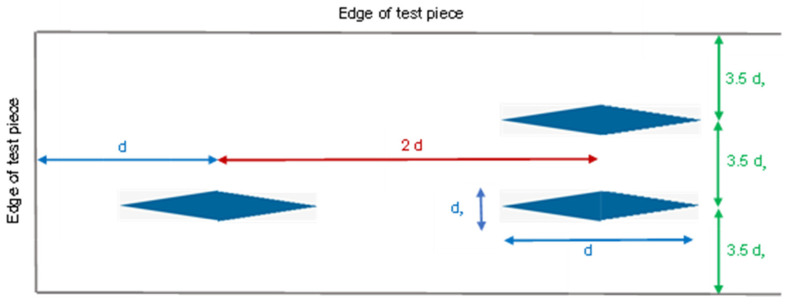
Minimum distance for Knoop indentation (ISO 4545-1:2023 standard [47]).

Surface hardness was evaluated using a Knoop hardness testing machine (FM-810, FUTURE-TECH, Kanagawa, Japan), measured 10 times, spaced 1 mm apart, and centered within the mold. The indentations were positioned approximately at least 1 mm from the mold’s edge. The ISO 4545-1:2023 [47] stipulates specific minimum spacing requirements for Knoop indentations. The center-to-center distance between any two indentations must be no less than twice the indentation’s diagonal (d) and three and a half times the indentation’s width (d_s_). Furthermore, the distance from the center of any indentation to the edge of the test piece must be at least equal to one diagonal (d) or three and a half times the indentation’s width (d_s_) (Figure 2). Surface hardness testing was performed by a single operator to maintain consistency and minimize variability in the measurement process.

A load of 50 g was applied to the specimens for intervals of 15 s, adhering to the guidelines outlined in ISO 4545-1:2023 [47]. Subsequently, the diagonal lengths of the indentations were measured using a stereomicroscope at 20× magnification. The Knoop Hardness Number (KHN) for each indentation was determined by measuring the indentation’s dimensions and applying the following formula: KHN = 14. 2 × (F/d^2^), where F = test load in Newtons; d = longer diagonal of an indentation in millimeters.

### 2.4. Statistical Analysis

The statistical analysis was performed with IBM SPSS. V29.0.1 (SPSS Inc., Chicago, IL, USA). Descriptive analyses were reported as mean and standard deviation. The normality of the data distribution was assessed using the Shapiro–Wilk test. The homogeneity of variance was determined by Levene’s test. A two-way ANOVA followed by Tukey’s post-hoc analysis was used to assess the effect of materials, GO concentrations, and their interactions on microhardness mean values. All tests are conducted at a significant level of *p* < 0.05.

### 2.5. Sample Size Calculations

G*power software (Version 3.1.9.7, Heinrich Heine Dusseldorf University, Dusseldorf, Germany) was used to determine the sample size. A total of 194 samples were found based on the pilot study (effect size f = 0.25, α err prob = 0.05, power (1 − β err prob) = 0.95, numerator df = 27, number of groups = 40). As a result, each group needs at least 14.2 samples. Therefore, fifteen samples per group were used in this investigation.

## 3. Results

**Figure 3:**
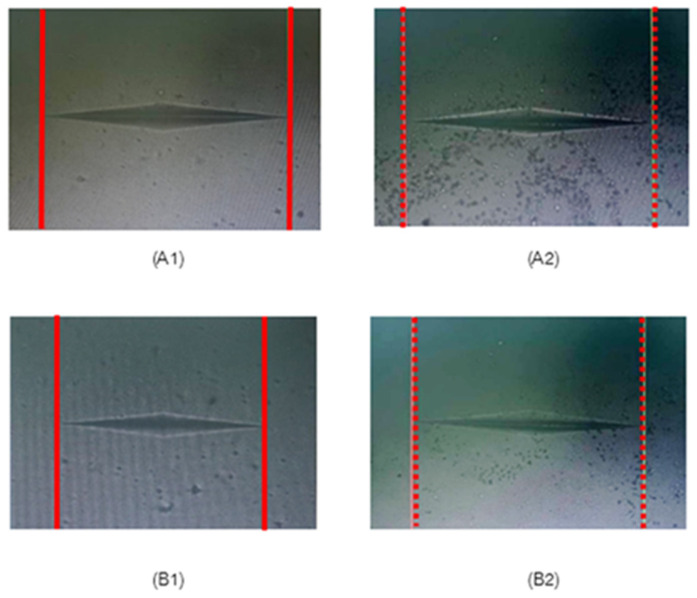
Stereomicroscope image at 20× magnification of control group and GO incorporated in experimental adhesives: Ada (control) (**A1**), 0.5 wt % GO incorporated in AdA (**A2**), OpA (control) (**B1**), and 0.5 wt % GO incorporated in OpA (**B2**).

Figure 3 illustrates a stereomicroscope image of GO integrated into the DAHs at 20× magnification. In terms of surface hardness, the indentation made in the control group was significantly wider than that observed in the 0.5 wt % GO group, indicating that the 0.5 wt % GO treatment enhances surface hardness relative to the control group (red line). As for the surface hardness data, Table 2 shows the mean KHN values of the different groups: control groups (two SCAs and two DAHs) and the experimental GO adhesives at varying concentrations (0.05 wt %, 0.1 wt %, 0.3 wt %, 0.5 wt %, 0.7 wt %, 1 wt %, 2 wt %, 5 wt %, and 10 wt %). The mean surface hardness values of the control groups were 26.15 ± 0.51 (ReG), 26.79 ± 0.83 (CoI), 17.89 ± 0.33 (AdA), and 29.81 ± 1.19 (OpA). In the control groups, the microhardness of OpA was significantly higher than that of the other groups (*p* < 0.05).

No significant differences were observed among the control, 0.05, 1, and 2 wt % GO concentrations within the ReG, CoI, and AdA groups (*p* > 0.05). Similarly, no significant differences were observed among 0.3–0.7 wt % GO concentrations across the same groups (*p* > 0.05). However, the groups incorporating GO exhibited significantly greater microhardness compared to their respective controls. At 0.5 wt % GO, the ReG group demonstrated a microhardness of 34.06 KHN versus 26.15 KHN in the control (*p* < 0.05). Similarly, the CoI and AdA groups showed significantly increased values of 33.73 KHN and 24.58 KHN, respectively, compared to 26.79 KHN and 17.89 KHN in their corresponding control groups.

There were no statistically significant differences observed among the control, 0.5, 0.7, and 1 wt % GO groups in OpA. Similarly, no significant differences were observed among the 0.05–0.3 wt % GO concentrations across the same groups (*p* > 0.05). Surface hardness was significantly increased (*p* < 0.05) when 0.05–0.3 wt %of GO was added to OpA compared to the control group. At 0.5 wt %, ReG, CoI, and AdA showed significantly higher microhardness compared to their respective control groups (*p* < 0.05). While in the 0.1 wt % groups, the microhardness of OpA was significantly higher than that of the other groups (*p* < 0.05). In the 1–10 wt % groups, the microhardness of ReG, CoI, and AdA demonstrated a gradual, significant decrease compared to the 0.7 wt % groups. Whereas in the 0.5–10 wt % groups, the microhardness of OpA showed a significant gradual decrease compared to the 0.3 wt % group (Table 2).

Table 3 presents the quantitative relationship between the percentage change in hardness and treatment concentrations relative to the control. An increase in hardness was observed within the concentration range of 0.05–2 wt % for ReG and AdA. For CoI, hardness increased between 0.05 and 1 wt %, while for OpA, the increase was observed within the range of 0.05–0.7 wt %. Microhardness values at GO concentrations between 0.3 and 0.7 wt % in ReG, CoI, and AdA were significantly higher than those observed in the control group (*p* < 0.05). In the OpA group, microhardness values at GO concentrations ranging from 0.05 to 0.3 wt % were significantly greater than those observed in the control group (*p* < 0.05). In contrast, a reduction in surface hardness was observed at elevated concentrations, specifically at 5–10 wt % for ReG and AdA, 2–10 wt % for CoI, and 1–10 wt % for OpA, with these values being significantly different from those of the control group (*p* < 0.05), indicating a potentially negative impact on material properties at higher treatment levels. An increase in surface hardness was observed at lower treatment concentrations; however, a gradual reduction was evident at higher concentrations.

**Figure 4:**
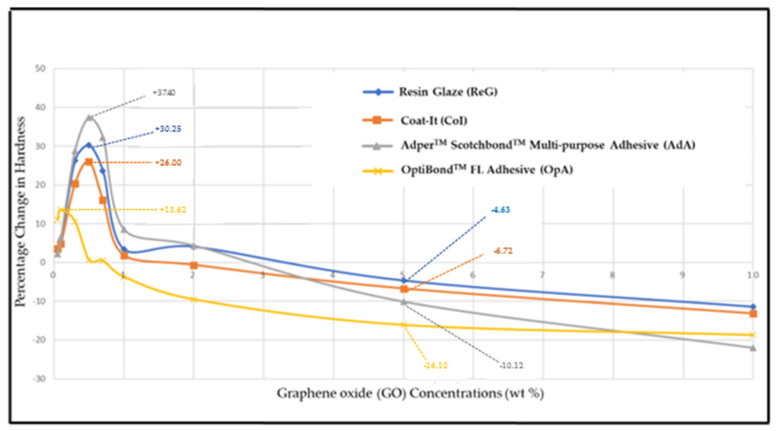
The graph illustrates the quantitative relationship between the percentage of changed hardness and concentrations relative to the control.

Figure 4 illustrates the quantitative relationship between GO concentration and the percentage change in hardness relative to the control group. The incorporation of GO resulted in marked enhancements in surface microhardness, with the most pronounced improvements observed at an intermediate concentration, specifically at 0.5 wt %. Notably, significant increases were recorded for ReG (34.06 vs. 26.15 KHN, +30%), CoI (33.73 vs. 26.79 KHN, +26%), and AdA (24.58 vs. 17.89 KHN, +37%) (*p* < 0.05), indicating the reinforcing effect of GO at optimal loading levels. OpA showed maximal improvement at 0.1 wt % (33.87 vs. 29.81 KHN, +13%). Significant hardness reductions occurred at higher concentrations: at 10 wt % GO, surface hardness decreased compared to the control by 11% in ReG (23.17 vs. 26.15 KHN), 13% in CoI (23.26 vs. 26.79 KHN), 22% in AdA (13.95 vs. 17.89 KHN), and 18% in OpA (24.23 vs. 29.81 KHN) (*p* < 0.05) (Table 3).

## 4. Discussion

The purpose of this study was to evaluate the impact of varying concentrations of GO combined with SCAs and DAHs on microhardness. We found that surface hardness was significantly increased in the GO and SCAs combinations in comparison to control samples (*p* < 0.05). The optimal concentration of GO was identified as 0.3–0.7 wt % for ReG, CoI, and AdA, while a lower optimal concentration of 0.1 wt % was observed for OpA. The incorporation of 0.5 wt % GO into AdA yielded a statistically significant enhancement in surface hardness, with an increase of up to 37% relative to the control group, while the addition of 0.1 wt % GO to OpA resulted in a 13% increase in surface hardness. Based on these results, the null hypothesis was rejected.

In this study, surface hardness was assessed using the Knoop microhardness test, following the ISO 4545-1:2023 standard [47]. Knoop indentation hardness testing offers superior measurement resolution due to the smaller width of the indentation (a pyramid-shaped diamond indenter). The shallow nature of the Knoop indentation makes it particularly suitable for evaluating the hardness of thin materials, especially in surface coatings. Moreover, when indentations are closely spaced or testing is at a specimen’s edge because of the indentation’s thin shape, the Knoop method is frequently employed. The precise placement of indentations during microhardness testing is critical. According to ISO 4545-1:2023 [47], the center-to-center distance between indentations must be at least twice the indentation diagonal and three and a half times the indentation width (Figure 2). In this study, ten indentations were made on each specimen, centered within the mold, with a consistent 1 mm spacing between centers. Microhardness measurements conducted in proximity to the margin of a workpiece frequently demonstrate more or less values, a phenomenon referred to as the edge effect. Thus, the indentations were positioned approximately at least 1 mm from the mold’s edge in this study to prevent the edge effect. Ning Hou et al. found that the microhardness value will be lower since the indentation site is so close to the edge of the workpiece. To minimize the influence of this phenomenon, they recommended that the distance between the indentation and the edge be no less than three times the indentation diagonal length under the applied load [48].

Among the SCAs and DAHs, both ReG and CoI, commercially available SCAs, are utilized to enhance the surface hardness of materials. Specifically, these agents demonstrated relatively high surface hardness values (Knoop): 26.15 ± 0.51 (ReG), 26.79 ± 0.83 (CoI), 17.89 ± 0.33 (AdA), and 29.81 ± 1.19 (OpA). Consequently, the disparate components within each of the SCAs and DAHs are shown to influence different surface hardness values. Both ReG and CoI are polymeric materials. The high surface hardness observed in these substances can likely be attributed to the presence of a significant concentration of multi-functional monomers. Variations in surface hardness can be attributed to differences in cross-link density and solvent content within the SCAs and DAHs. The degree of cross-linking, which influences the material’s rigidity, and the presence or absence of solvents, which can affect the material’s structure and interaction with the substrate, both play a role in determining the final surface hardness. OpA demonstrated a significantly superior surface hardness compared to AdA. This superior performance is attributed to OpA’s composition, which includes 48 wt % barium glass filler [49].

Numerous studies have demonstrated that GO can significantly enhance the durability and strength of dental materials [38,39,40,41]. The durability and abrasion resistance of dental materials are crucial for both temporary and prosthetic applications due to the constant occlusal forces experienced during chewing. GO has been found to be more capable of altering the tribological characteristics [50]. Ramanathan et al. found that the modulus of elasticity, mechanical resistance to compressive strength, and wear resistance increased significantly following the addition of graphene [51]. Abrasion resistance increased by 14.76% with the addition of 0.1 wt % GO [52]. The incorporation of 0.7 wt % GO, which exhibits superior mechanical properties, led to a 64.89% increase in mechanical performance compared to the control group, thereby significantly improving both the mechanical properties and durability of the coating [53]. According to our study, the optimal GO concentration range was 0.3–0.7 wt % for ReG, CoI, and AdA, with statistically significant hardness increases of 30% (34.06 vs. 26.15 KHN), 26% (33.73 vs. 26.79 KHN), and 37% (24.58 vs. 17.89 KHN), respectively (*p* < 0.05). For OpA, the maximal improvement (13% increase, 33.87 vs. 29.81 KHN) occurred at 0.1 wt %, significantly lower than other materials (*p* < 0.05). A previous study suggested that a small amount of GO can significantly increase the material’s strength in resin-based dental materials, and that GO content varies between 0.024 and 2.52 wt/wt [42,43]. Even at the lowest GO concentration of 0.024 wt/wt, significant improvements in mechanical properties were observed [44,45]. Similar to Mehran T. et al., the addition of functionalized graphene oxide (fGO) nanosheets to acrylic resin was found to significantly improve the surface hardness of the composite material. Their study revealed that a 0.25 wt % addition of GO led to a substantial increase in surface microhardness [54]. Moreover, Khan et al. demonstrated that incorporating 0.024 wt % and 0.048 wt % GO into a polymethyl methacrylate (PMMA) matrix significantly enhanced both the abrasion resistance and flexural strength of the resulting composite material [45]. The small 1% graphene nanosheet addition to PMMA significantly enhanced its mechanical properties, leading to a 20% increase in strength and an 80% increase in the modulus of elasticity.

However, other studies have reported slight effects of adding graphene to dental materials. Punset et al. investigated the impact of graphene on PMMA discs used for removable and fixed dental prostheses [55]. They found that adding 0.1027 wt % graphene to PMMA did not significantly increase the Vickers hardness number (HVN), with values of 23.6 HVN for PMMA and 24.6 HVN for graphene-reinforced PMMA [55]. Similarly, Lee et al. reported a slight increase in surface hardness with the addition of 0.5 wt % graphene to PMMA; however, this was not deemed sufficient for clinical use due to the lack of chemical bonding between the graphene and the polymer matrix. This study demonstrated that a statistically significant decrease in surface hardness was observed at GO concentrations exceeding 5 wt % in ReG and AdA, 2 wt % in CoI, and 1 wt % in OpA. At a GO concentration of 10 wt %, notable decreases in surface hardness were recorded in all experimental groups, with reductions of 11%, 13%, 22%, and 18% for ReG, CoI, AdA, and OpA, respectively (*p* < 0.05). An increase in surface hardness was observed at lower treatment concentrations; however, a gradual reduction was evident at higher concentrations. A previous study also found that higher graphene concentrations (1 wt % and 2 wt %) actually decreased the flexural strength and hardness of PMMA [56]. Wang et al. demonstrated that a further increase in GO concentration to 2 wt % resulted in a decline in the mechanical properties of composite material [57]. Alvaredo-Atienza observed that adding 10% graphene nanoplatelets (GNPs) to polyetheretherketone (PEEK) reduced its flexural strength [58]. Consistent with our findings, the incorporation of 10 wt % GO in DAHs resulted in a significant reduction of up to 22% in surface hardness. These findings suggest that while graphene can improve certain properties of dental materials, high concentrations may lead to reduced mechanical performance. In this study, it was possible to estimate the highest concentration of GO incorporated into SCAs that would not result in a decrease in surface hardness. This suggests that there is an optimal concentration at which GO integration enhances the mechanical properties of adhesive coatings. The mechanical behavior of a composite is critically determined by the uniformity of its composition and the strength of the bond between the nanomaterials and the polymer matrix. Excessive incorporation of GO into the resin matrix can adversely affect the mechanical performance of nanocomposite materials due to several interrelated phenomena. At elevated concentrations, GO nanosheets exhibit a strong tendency to restack, forming multilayered graphite-like aggregates as a result of van der Waals forces and π–π interactions between adjacent sheets. This restacking behavior significantly reduces the homogeneity of dispersion and limits the effective surface area of GO available for interaction with the surrounding polymer matrix, thereby diminishing its reinforcing efficiency [59]. Additionally, excessive GO loading promotes agglomeration, which not only disrupts the structural integrity of the composite but also increases the viscosity of the mixture, negatively impacting processability and mechanical behavior. Although graphene’s intrinsic two-dimensional structure and exceptional mechanical properties offer considerable potential for reinforcement, these benefits are compromised when GO is present in excess. The observed reduction in surface microhardness at higher GO concentrations in this study may be attributed to these factors. Consequently, the precise control and optimization of the GO concentration are essential to maximize its reinforcing potential while avoiding adverse effects on the structural and functional properties of the adhesive system. Therefore, careful optimization of GO concentration is essential to fully harness the benefits of graphene without compromising the material’s strength.

The dispersion of GO within the resin matrix is crucial for the effectiveness of the filler. Achieving the uniform distribution of GO can be challenging; however, methods like sonication, mechanical stirring, and surfactants are commonly used to improve dispersion. In this study, the experimental adhesive was centrifuged to achieve a homogeneous dispersion of GO, followed by sonication for one hour to ensure even mixing. According to Paz et al., ultrasonication is particularly effective in uniformly dispersing GO particles within a polymer matrix, leading to a more consistent and durable composite material [60]. This study demonstrated that intermediate concentrations of GO led to significant improvements in surface hardness (0.3–0.7 wt %), likely due to the formation of a well-dispersed reinforcing network within the resin matrix of both SCAs and DAHs. Such dispersion enhances stress transfer efficiency and minimizes defect propagation, thereby optimizing the mechanical performance of the adhesive systems. For example, AdA is an unfilled adhesive system composed primarily of Bis-GMA and formulated without solvent. The absence of inorganic fillers results in a higher proportion of resin matrix, yielding a more homogeneous environment that likely facilitates uniform dispersion of GO across a wider range of concentrations. This compositional characteristic may explain the observed enhancement in microhardness at GO concentrations between 0.3 and 0.7 wt %, as the resin-rich matrix can accommodate and integrate greater amounts of nanofiller without inducing phase separation or agglomeration. In contrast, OpA contains a high-filler loading of approximately 48% by weight, composed of barium glass particles with an average diameter of 0.6 µm. This substantial inorganic content reduces the relative volume of the resin matrix, which primarily consists of Bis-GMA and TEGDMA. The limited resin phase in such a densely filled system may restrict the capacity for effective GO dispersion at higher concentrations. However, at lower concentrations—specifically 0.1 wt %—GO may be more uniformly distributed, enabling the formation of a well-integrated reinforcing network that enhances mechanical properties such as surface hardness and resistance to deformation. These findings underscore the importance of adhesive matrix composition in modulating the optimal concentration range for nanofiller incorporation and suggest that the filler-to-resin ratio plays a critical role in determining the efficiency of GO reinforcement.

The observed reduction in microhardness at elevated GO concentrations is hypothesized to result from the agglomeration of graphene oxide within the adhesive matrix, as suggested by prior studies. However, direct morphological evidence to support this hypothesis was not obtained in the present study, as characterization techniques such as scanning electron microscopy (SEM) or transmission electron microscopy (TEM) were not employed. Future investigations are warranted to incorporate such analyses to verify the dispersion state of GO and to elucidate its influence on the mechanical properties of the adhesive materials.

The selection of ethanol as a solvent for GO powder is significant, as it is consistent with previous research by Paredes et al. This research highlighted the long-term stability of various organic solvents, including ethanol, compared to aqueous solutions when subjected to sonication for one hour and centrifugation at 500× *g* rpm for 90 min. Experimental findings indicate that GO exhibits a reasonable degree of solubility in ethanol [61].

The incorporation of graphene into dental materials demonstrates considerable clinical potential, particularly in enhancing the mechanical durability and service life of dental prostheses and dentures. These improvements may contribute to better clinical outcomes and overall oral health-related quality of life for patients. Nonetheless, the application of graphene-based materials may be limited in certain clinical scenarios. Specifically, in cases where high aesthetic demands are required, the addition of graphene may be unsuitable due to the dark coloration it produces at concentrations exceeding 0.35 mg/mL [62]. Furthermore, concentrations of graphene oxide (GO) at or above 50 µg/mL have been reported to exert cytotoxic effects, including damage to human fibroblasts and T lymphocytes [63,64]. Consequently, further research is necessary to thoroughly evaluate the long-term clinical performance and biocompatibility of GO-reinforced dental materials.

The limitation of this study is that it was performed in vitro. There are currently no reports of the potential long-term toxicity of graphene in the oral cavity. Further study is needed to investigate the long-term effects of dynamic pH changes, which simulate the oral condition, on the mechanical properties of graphene-reinforced composites. Another limitation of this study is that, to minimize oxygen exposure and prevent the formation of an oxygen-inhibited layer, both control and experimental GO adhesives were dispensed into resin molds and covered with a Mylar strip, with all specimen preparation performed within a nitrogen-purged chamber. However, the formation of the oxygen-inhibited layer on the surface coating could not be effectively controlled under clinical conditions, which may have influenced the polymerization behavior and surface properties of the tested materials.

## 5. Conclusions

The incorporation of GO at carefully controlled concentrations led to significant enhancements in the microhardness of SACs (ReG and CoI) and DAHs (AdA and OpA). The most pronounced improvements were observed at GO concentrations between 0.3 wt % and 0.7 wt % for ReG, CoI, and AdA, whereas OpA exhibited optimal performance within the lower concentration range from 0.05 wt % to 0.3 wt %. In contrast, higher GO concentrations (≥1 wt %) were associated with a progressive decline in microhardness across all adhesive systems. This concentration-dependent trend indicates that surface hardness increases at lower GO concentrations, likely due to uniform dispersion, but diminishes at higher loadings, possibly due to particle agglomeration and the disruption of the matrix structure. These findings clearly demonstrate the potential of GO as an effective nanofiller for enhancing the surface mechanical properties of dental adhesive systems. The results support its promising application in restorative dentistry, though additional studies are recommended to validate long-term performance and clinical outcomes.

## Figures and Tables

**Table 1:** Materials used in this study.

Materials	Compositions
Graphene oxide	Carbon atom (synthesis using Modified Hummers’ method)
Resin Glaze (Shofu Inc., Kyoto, Japan) Lot: 082246	Multi-functional monomers, methacrylate monomers, Others
Coat-It (Shofu Inc., Kyoto, Japan) Lot: 042206	Multi-functional monomers, MMA, phosphonic acid monomers, polymerization initiators, and others
Adper^TM^ Scotchbond^TM^ Multi-purpose Adhesive (3M EPSE, Seefeld, Germany) Lot: NF40227	Adhesive: Bis-GMA, HEMA, tertiary amine, photoinitiator
OptiBond^TM^ FL Adhesive (Kerr Corporation, Orange, CA, USA) Lot: A158978	Adhesive: TEGDMA, UDMA, GPDM, HEMA, Bis-GMA, barium glass, ethanol, water, camphorquinone, tertiary amine

Abbreviations: MMA, methyl methacrylate; Bis GMA, bisphenol-A-glycidyl-methacrylate; HEMA, hydroxyethyl methacrylate; TEGDMA, triethylene glycol dimethacrylate; UDMA, urethane dimethacrylate; GPDM, glycerophosphate dimethacrylate.

**Table 2:** Mean and standard deviation of microhardness values (Knoop) (n = 15) in the experimental group.

Surface Coating Agents (SCAs) and Dental Adhesives (DAHs)	Concentration of GO (wt %)									
	Control	0.05	0.1	0.3	0.5	0.7	1.0	2	5	10
Resin Glaze (ReG)	26.15 ± 0.51 ^(a,1)^	26.92 ± 0.34 ^(a,1)^	27.61 ± 0.60 ^(a,1)^	33.02 ± 0.55 ^(b,1)^	34.06 ± 0.47 ^(b,1)^	32.34 ± 0.49 ^(b,1)^	27.04 ± 0.40 ^(a,1)^	27.22 ± 0.13 ^(a,1)^	24.94 ± 0.20 ^(c,1)^	23.17 ± 0.75 ^(c,1)^
Coat-It (CoI)	26.79 ± 0.83 ^(a,1)^	27.73 ± 0.86 ^(a,1)^	28.08 ± 0.50 ^(a,1)^	32.25 ± 0.92 ^(b,1)^	33.73 ± 0.74 ^(b,1)^	31.11 ± 0.49 ^(b,1)^	27.28 ± 0.65 ^(a,1)^	26.62 ± 0.81 ^(a,1)^	24.99 ± 0.57 ^(c,1)^	23.26 ± 0.59 ^(c,1)^
Adper^TM^ Scotchbond^TM^ Multi-purpose Adhesive (AdA)	17.89 ± 0.33 ^(a,2)^	18.29 ± 0.48 ^(a,2)^	19.05 ± 0.16 ^(a,2)^	23.04 ± 0.68 ^(b,2)^	24.58 ± 0.36 ^(b,2)^	23.67 ± 0.73 ^(b,2)^	19.41 ± 0.58 ^(a,2)^	18.67 ± 0.59 ^(a,2)^	16.08 ± 0.35 ^(c,2)^	13.95 ± 0.48 ^(d,2)^
OptiBond^TM^ FL Adhesive (OpA)	29.81 ± 1.19 ^(a,3)^	33.12 ± 0.73 ^(b,1)^	33.87 ± 1.11 ^(b,3)^	32.97 ± 0.63 ^(b,1)^	30.01 ± 0.36 ^(a,3)^	29.95 ± 0.23 ^(a,3)^	28.71 ± 0.47 ^(a,1)^	26.97 ± 0.29 ^(c,1)^	25.01 ± 0.54 ^(d,1)^	24.23 ± 0.71 ^(d,1)^

The same lowercase superscript letter (in rows) indicates mean values without statistically significant differences (*p* > 0.05). The same number (in columns) indicates mean values without statistically significant differences (*p* > 0.05).

**Table 3:** Quantitative relationship between the percentage change in hardness (%) and treatment concentrations relative to the control.

Surface Coating Agents (SCAs) and Dental Adhesives (DAHs)	Concentration of GO (wt %)								
	0.05	0.1	0.3	0.5	0.7	1.0	2	5	10
Resin Glaze (ReG)	+2.94	+5.60	+26.27	+30.25	+23.67	+3.40	+4.09	−4.63	−11.4
Coat-It (CoI)	+3.51	+4.82	+20.38	+26.00	+16.12	+1.83	−0.63	−6.72	−13.18
Adper^TM^ Scotchbond^TM^ Multi-purpose Adhesive (AdA)	+2.24	+6.5	+28.78	+37.4	+32.31	+8.5	+4.36	−10.12	−22.02
OptiBond^TM^ FL Adhesive (OpA)	+11.10	+13.62	+10.60	+0.67	+0.47	−3.70	−9.53	−16.10	−18.72

An increase in hardness relative to the control is reflected by positive values, whereas negative values correspond to a reduction in hardness.

## Data Availability

The original contributions presented in the study are included in the article; further inquiries can be directed to the corresponding authors.
